# A method for evaluating cognitively informed micro-targeted campaign strategies: An agent-based model proof of principle

**DOI:** 10.1371/journal.pone.0193909

**Published:** 2018-04-10

**Authors:** Jens Koed Madsen, Toby D. Pilditch

**Affiliations:** 1 School of Geography and the Environment, University of Oxford, Oxford, United Kingdom; 2 Department of Experimental Psychology, University College London, London, United Kingdom; University of Vermont, UNITED STATES

## Abstract

In political campaigns, perceived candidate credibility influences the persuasiveness of messages. In campaigns aiming to influence people’s beliefs, micro-targeted campaigns (MTCs) that target specific voters using their psychological profile have become increasingly prevalent. It remains open how effective MTCs are, notably in comparison to population-targeted campaign strategies. Using an agent-based model, the paper applies recent insights from cognitive models of persuasion, extending them to the societal level in a novel framework for exploring political campaigning. The paper provides an initial treatment of the complex dynamics of population level political campaigning in a psychologically informed manner. Model simulations show that MTCs can take advantage of the psychology of the electorate by targeting voters favourable disposed towards the candidate. Relative to broad campaigning, MTCs allow for efficient and adaptive management of complex campaigns. Findings show that disliked MTC candidates can beat liked population-targeting candidates, pointing to societal questions concerning campaign regulations.

## Introduction

Political campaigns aim to influence the beliefs of the electorate to make them more likely to vote for their preferred candidate. In most free elections, engagement between candidates and voters is an essential component of the election persuasion process. Aside from the persuasiveness of argument content, the voter’s perception of the candidate’s credibility appears integral to the success of engagement (e.g. [1]). If the voter believes the candidate is a liar, the persuasive attempt may fail regardless of the content of the argument (indeed, a liar may be expected to present extraordinarily good ‘data’ for their position, as liars are unbound by the constraints of empirical veracity). A message may even provoke a backfire effect (e.g. [2]-[3]) where an endorsement of a policy makes the policy seems less believable to a voter with strong negative beliefs about the credibility of the source.

Individual profiling is increasingly prevalent in political and other campaigns [4]-[5]. For example, President Obama’s campaign used personalized ads through social media in 2008 and 2012 [6]. These campaigns are known as ‘micro-targeted campaigns’ (MTCs). Given relevant data of the electorate, campaigns may infer possible political affiliation, the voting likelihood, personality traits, preferred political issues (e.g. the economy, social policies, etc.), and so forth. Big data allows for highly specified models of each individual voter, which allows for targeted political adverts that address specific political issues in a way that is tailored to the individual in question.

While MTCs have become more frequent (and will presumably become even more so in years to come), it remains open whether they are, in fact, an effective way of campaigning in a dynamic political environment. Further, if they *are* effective tools of campaign management, it is unclear *how* effective they are compared with population-targeted campaign strategies. Critically, it is also not clear how to best test the efficiency of MTCs (e.g. amount of money spent per vote, the degree to which persuasive messages manage to change votes, time spent per vote, etc.). To our knowledge, the current paper is the first to propose a formal method for exploring the efficiency of micro-targeted campaign strategies.

The paper provides a cognitive and methodological framework for simulating MTCs in dynamic environments over a period of time. In exploring these questions, we use cognitively informed agents in an Agent-Based Model (ABM). We hypothesise that MTCs are effective ways of adaptively managing complex campaign situations. Increasingly accurate and complete information about the voters may inform outreach strategies change the nature of campaigning from broad and stochastic communications to increasingly targeted and specified messages.

More broadly, we argue that ABMs are a useful tool for implementing, testing, and analysing cognitive models in dynamics, complex systems, where cognitive functions can be encoded into agents, whom then interact with each other and their environment. Social networks have been shown to be important in moderating core political attitudes [7], in belief and moral formation [8], and in persuasion [9].

MTCs can be used in different domains (political campaigns, public health campaigns, etc.). Consequently, each situation may require different cognitive functions. The purpose of the present paper is two-fold: First, we seek to provide a proof of principle of the impact of MTCs, inserting sensible cognitive architecture on the part of individuals into a novel, dynamic societal level simulation. Second, in forwarding this proof of principle, we aim to plant a banner for the potency of using cognitively informed ABMs to address issues beyond the individual. This allows for simplification of cognitive principles and a pure test of the methodology of an Agent-Based Model of MTCs.

### Agent-based models

Analytic computational models typically describe cognitive functions either in isolation (e.g. belief revision given new information, [10]) or in dyadic relations (e.g. prisoners’ dilemma, [11]). However, when agents interact and influence each other spatiotemporally, behaviour may become dynamic and adaptive, resulting in computationally irreducible systems. Behaviours in irreducible systems cannot be predicted given the base system state alone. For this reason, predictions of individual and aggregate behaviour in complex systems are difficult or impossible with analytic models [12].

Agent-Based Models (ABMs) are computer simulated multi-agent systems that describe the behaviour of and interactions between individual agents who operate in synthetic environments [13]–[15]. They allow for adaptive strategies such that agents can change behaviour given changes in information of context [16]. This is essential for developing a method to test adaptive campaign management strategies. As cognitive models are computational in nature, ABMs can integrate them within a complex system to test the epistemic and behavioural patterns emerge as a consequence of interaction between computational models across space and time.

ABMs typically contain three elements: patches, agents, and links. Patches typically represent the physical environment in which agents act. However, as contact between candidates and voters can occur regardless of distance, physical dimensions are irrelevant in the current model. Consequently, the following focuses on *agents* and *links*.

#### Agents

The entities that act in the simulated world. The cognitive make-up of agents can be any rule that constrains or enables behaviour within the simulated world. For example, agents may revise their beliefs about the world by interacting with and gaining information from the environment. Along with cognitive functions, agents can have physical functions such as motion, energy consumption, and aging. Agents may differ in both the specifics of their behaviours, and the parameters gained from them. This allows for heterogeneity within an agent population, and thus the capacity to model real world populations with greater ecological validity.

#### Links

Typically representing interaction rules between agents, links may be encoded with functional capacities. These can be any and all social links that inform and influence behaviour. Such interaction is often essential in the development and representation of system complexity. Links can be direct (e.g. providing information to another agent) or indirect (e.g. some agents might prefer to be in the vicinity of other types of agents). In the current model, only direct links are employed, as the persuaders contact voters directly. These links are historical representations of campaigns having made contact with a voter.

ABMs are ideally suited to explore and test the effectiveness of (political) campaign strategies for three reasons. First, candidates and campaign managers are capable of adapting to changes in the political milieu and altering their strategies or target groups as new information emerges. ABMs can incorporate adaptive strategies in dynamic environments, as agents can react and respond to new information or changes in the population. In the present model, campaigners engage with voters as the campaign unfolds. Second, given these spatiotemporal interactions, campaigns are complex and therefore computationally irreducible. As mentioned above, ABMs provide an ideal tool for overcoming such irreducibility. Third, ABMs allow for a heterogeneous population (here, the electorate and the campaigners). Some may have an inherent dislike for government or specific candidates, and voters are more or less inclined to actually vote. This is captured in the present model by having voters differ in their subjective perception of the credibility of each candidate.

While relatively new in cognitive sciences, ABMs have previously been used to account for related cognitive phenomena such as belief diffusion in networks [17], opinion dynamics [18], the emergence of conspiratorial thinking in social networks [19], attitude change [20], and belief cascading [21].

The current model relies on three functions: how voters perceive the source credibility of each candidate, how voters revise their beliefs when candidates engage with them, and the strategy of engagement.

### Source credibility in politics

Source credibility influences human cognitive phenomena related to political campaigns. It affects reception of persuasive messages [22], [23], impacts juror decision making [24], increases adherence with persuasion strategies [25], and influences how people are seen in and can influence social situations [26]-[27].

Additionally, credibility increases public policy compliance [28], influences candidate choice [29], increases intention of voting [30], though not necessarily actual voting [31], and increases societal cooperation [32]. Lack of trust may instigate civic participation [33]. Given the importance of source credibility in politics, we have chosen to take candidates’ credibility as the cognitive framework for providing a methodological proof of principle of the MTCs.

Credibility influences the degree to which people are persuaded by a message. However, there is considerable disagreement in the literature concerning the descriptive and normative functions of credibility in persuasion and argumentation. The Elaboration-Likelihood Model (ELM [34]) and Heuristic-Systematic model (HSM [22]) describe the source of a message as a shallow heuristic rather than an analytic cue [35]. Comparatively, Bayesian models integrate credibility in revising beliefs when given evidence from a source [10], [36]-[37]. In the view of the former, the nature of the source should matter less when given an incentive to critically analyse the content of the argument. In the latter view, the credibility of the source remains an important part of belief revision regardless of the level of concentration.

Here, we employ the Bayesian source credibility model for three reasons. First and foremost, compared with the directional predictions of ELM and HSM, Bayesian models provide computationally specific and implementable functions. Predictions in the ELM and HSM are directional and reliant on message elaboration. On this view, people should increasingly disregard the source of the message (credible or not) if they invest more cognitive effort in elaborating on the rational structure of the message and its evidence. Directionally, sources are seen as shallow cues that increase adherence when people do not elaborate and think carefully. For the purpose of modelling influence of source credibility, this relies on assumptions concerning message elaboration, the function of which is underspecified. For this reason, a Bayesian model is easier to implement, as the functions are fully computationally specified within the model. Second, the Bayesian model enjoys a good fit with predictions concerning the influences of source credibility in political argumentation [1] and with belief revision more generally [10]. Finally, while other factors may also influence political belief dynamics, these are typically bound to specific political contexts and cultures. While situational, economic, cultural, and global factors have fluctuating importance in specific elections, the potential impact of the candidates themselves is constant. As cited above, credibility is a foundational component of reasoning and political campaigning more generally. As such credibility is an ideal choice for a proof of methodological principle of MTC campaigns. In choosing a well-tested Bayesian source credibility model, we gain a computationally specified model that can be implemented in an ABM. In the following, we present this model.

### Belief revision: Bayesian updating given testimony

Bayesian reasoning take point of departure in subjective, probabilistic degrees of beliefs in propositions where Bayes’ theorem captures the posterior degree of belief given a prior belief in the hypothesis and some new evidence [38]. The approach has been applied to argumentation theory [39]-[40] and suggests that Bayesian reasoning may account for crucial elements of human information integration in practical reasoning. Most relevant to the current model, a Bayesian model of source credibility describes how humans *should* integrate uncertain information from more or less reliable sources [36]. The model has a good fit with observed responses [1], [10], suggesting that the model also describes how people *do* integrate information from more or less credible sources.

**Fig 1:**
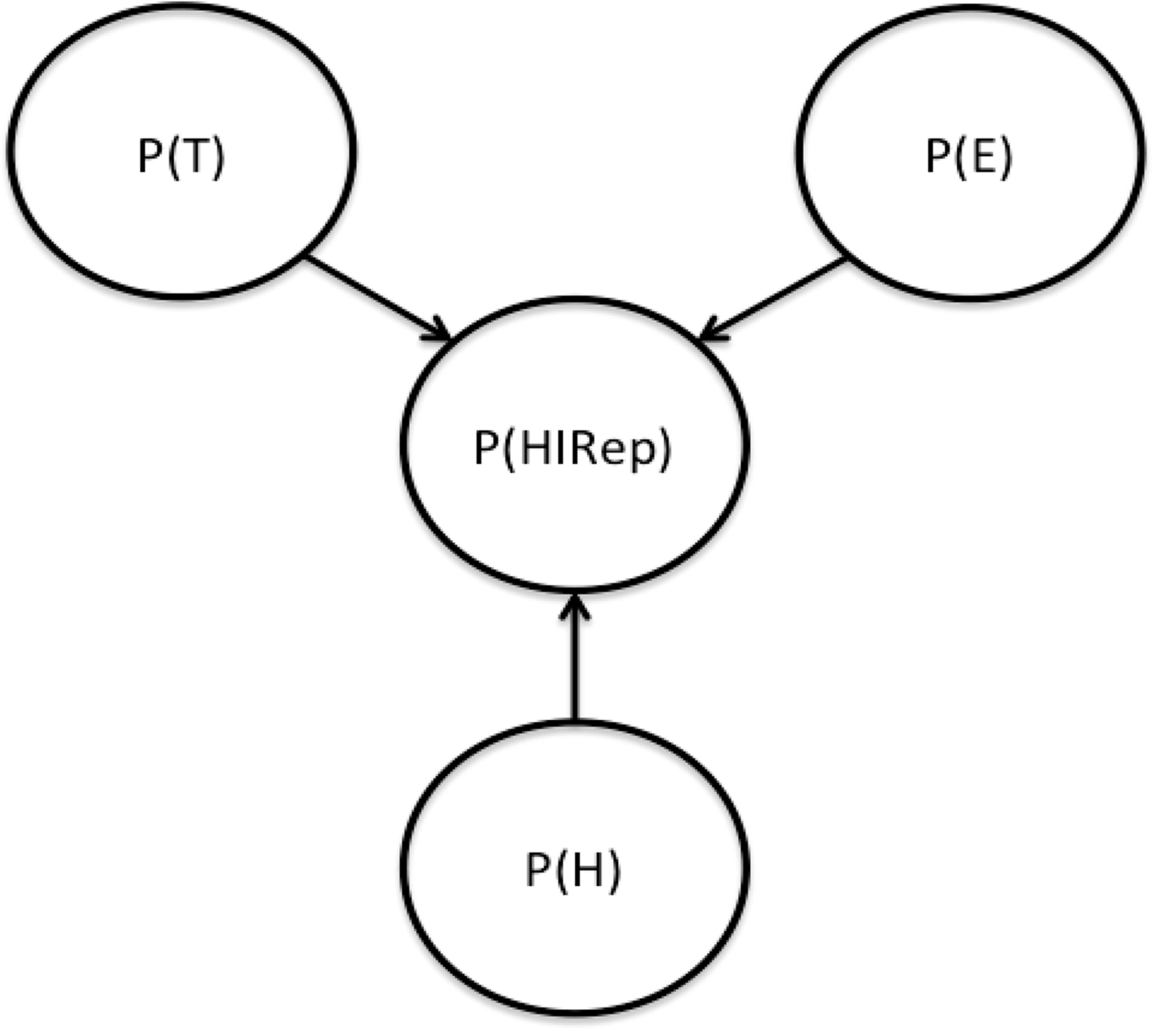
A Bayesian source credibility model.

In this model, credibility is defined as a combination of trustworthiness and expertise ([37], see also [10], Fig 1). In order to implement the model and facilitate communication between persuaders (candidates) and persuadees (voters), the members of the electorate have subjectively estimated beliefs about the credibility of each persuader.

Perceived *expertise*, P(E), refers to the persuader’s likely **capability** of providing accurate information. Perceived *trustworthiness*, P(T), refers to the persuader’s likely **intention** of providing accurate information. Expertise and trustworthiness are orthogonal and independent. In the model, each voter has subjective perceptions of a candidate’s P(E) and P(T)

To calculate voters’ belief revision, we employ an expanded version of Bayes’ theorem that incorporates perceived trustworthiness and expertise. The computational model is taken from [10] and relies on advances made in [36] and [37] (see also [41]).

$$p(h|rep)\;=\;\frac{p(h)p(rep|h)}{p(h)p(rep[h)+p(\neg h)p(rep|\neg h)}$$ (1)

P(h|rep) represents the probability that the hypothesis is true (h) given a confirming statement (rep). P(h) represents prior belief in the hypothesis, and p(rep|h) and p(rep|¬h) represent the conditional probability that the source would provide a positive statement if the hypothesis was indeed true/false. Trustworthiness and expertise are integrated within P(rep|h) and P(rep|¬h) through the combination of conditional probabilities (see [10] for formal description).

P(E) and p(T) represent prior beliefs in expertise and trustworthiness. These differ heterogeneously within the voter population (μ = 0.5; σ = 0.25 for both). The conditional probability table represents the epistemic relationship between model parameters and the likelihood of providing true or good advice. For example, p(Rep|H, E, T) refers to the likelihood that a speaker would declare a hypothesis to be true when the speaker has *complete and perfect knowledge* of the topic, is *completely trustworthy* in a world where the hypothesis happens to be *true* regardless of the statement of the speaker. We adopt the conditional table (Table 1) from [1] who extracted empirical conditional parameters from US voters.

**Table 1:** Conditional probability table.

	T, E	T, ¬E	¬T, E	¬T, ¬E
**H**	0.80	0.58	0.34	0.18
**¬H**	0.22	0.42	0.59	0.71

In summary, the Bayesian model of source credibility provides computationally specific and parameter-free belief revision such that the agent makes use of its estimation of the persuader’s source credibility to update its belief when the persuader contacts the voter with a persuasive statement. Along with the modelling advantage of having a fully specified computational model, the Bayesian model, as previously mentioned, enjoys a good fit with observed data of how people integrate information from more or less credible sources.

## An agent-based model of micro-targeted campaign strategies: Method and model description

### Method

In order to explore the effect of MTCs in principle, we simulate an election campaign through an ABM in which the candidates (the persuaders) can interact with the voters (the persuadees). Though exploratory in nature, the model has two aims. First, to our knowledge, although some models have explored opinion change in politics (e.g. [17]), ABMs have not been used to directly explore campaign strategies. The paper provides a novel method for exploring the efficiency of persuasion campaign strategies. Typically, a cognitive approach to political reasoning would involve data collected from participants, necessitating a more traditional methods section with design, procedures, participant descriptions, and materials. However, the aim of the paper is to provide a novel method for exploring cognitive assumptions and campaign strategies in a complex environment. Consequently, we employ stylised cognitive components (see description below) in an agent-based model (ODD+D protocol [42] and model code is available upon request from either author, see S1 File). Typically, an ABM describes model parameters (including cognitive function and interactions) and subsequently run simulations to test the strength of the manipulation. Second, we explore the relative efficiency of MTC campaigning when voters consider the credibility of the source is explored. We run 3x3x10 simulations where the average credibility for each candidate is varied (μ_cred_ = 0.4; 0.5; 0.6) and 10 different reach parameters. Complex systems are probabilistic in nature, as the end-state of each simulation will vary slightly from the others (if the system is unstable, the end-states will vary greatly, if the system is stable, the results will vary little). To smooth simulation trends, each combination of simulations was run 100 times, yielding 9000 simulations (9 different simulations, each run 100 times with 10 different reach parameters). In the results section, we report the average outcomes for each simulation. The model was developed in NetLogo v. 5.2.1

### Model description

The model has three types of agents: Voters (recipients), a candidate using a micro-targeted campaign (MTC), and a candidate using a stochastic campaign (non-MTC). For clarity, ‘stochastic’ refers to campaign strategies that do not differentiate between voters, but will seek contact with any voter indiscriminatingly. For example, a political ad on a bus communicates broadly to anyone within a particular city, but does not directly target specific voters. These are described in the following.

When given statements from candidates, voters revise their beliefs in accordance with the Bayesian source credibility model. When setting up the model, each voter generates an expertise and trustworthy score for each candidate from a normal distribution (as described later, we manipulate the means in the two simulations, such that μ = 0.4, 0.5, or 0.6, σ = 0.25). To fully parameterize the model, voters have the above conditional probability table (Table 1). This allows for belief revision given persuader statements with no free parameters. Further the standard deviation yields voter heterogeneity, as one voter may rate a candidate as highly credible while another voter rates the same candidate poorly. To provide a ‘signalling’ factor for the MTC candidate, voters average trustworthiness and expertise scores to generate a ‘credibility score’. The Bayesian model motivates this signalling score, as credibility is defined as an amalgamation of expertise and trustworthiness.

Voters use their belief in the hypothesis (P(candidate)) to determine their eventual vote. If a voter has p(candidate) < 0.5, it favours the non-MTC candidate; if p(candidate) > 0.5, it favours the MTC candidate. When setting up the model, each voter generates a P(candidate) value from a normal distribution (μ = 0.5, σ = 0.25, bounded between 0.01 and 1), representing a voter’s initial (prior) preference between the candidates. Each voter similarly generates a voting likelihood (P(vote); μ = 0.5, σ = 0.25, bounded between 0.01 and 1). The campaign runs for 50 days (50 ticks). At the end of the simulation, voters vote (with a likelihood dictated by P(vote)) for their favoured candidate (dictated by P(candidate)). There are 10,000 voters in the simulation.

Both persuaders’ aim to shift voters p(candidate) towards their own position. In order to do so, they establish connection with voters and make opposite claims. In accordance with the source credibility model, the non-MTC candidate represents p(candidate) = 1 while the MTC candidate represents p(candidate) = 0. This gives full implementation of the Bayesian source credibility model where the voter updates the prior belief given representation by a (more or less) credible source. After each connection, the contacted voter takes P(candidate|rep)–i.e. the posterior—as their new value for p(candidate)

For each tick, the candidate can establish contact with X voters, defined as ‘candidate reach’. In the simulations, the MTC candidate has a fixed reach of 20 the reach of the non-MTC candidate is manipulated to test the efficiency of the MTC strategy. In the below visualisation of the simulations, the reach ratio is the reach of the non-MTC divided by the reach of the MTC.

Central to the model, the MTC and non-MTC campaigns differ in their contact selectivity. The non-MTC is fully stochastic and thereby corresponds to a blind campaign that distributes leaflets or conducts cold-calls with no knowledge of the electorate. The MTC segments voters and only contacts those who are not already strongly for or against the candidate, and perceive the candidate as credible, and are likely to vote. That is, P(candidate) > .25 & < .75, P(candidate_cred_) > = .5 and P(vote) > .5. In practical terms, this means the MTC candidate does not spend energy on voters who strongly oppose her candidacy (P(candidate) > .25) or misuse energy contacting voters who already strongly support her (P(candidate) < .75). The signalling factor ensures that the candidate only contacts voters who will be willing to engage with her persuasive message (P(candidate_cred_) > = .5). Finally, the MTC contacts voters who are likely to actually vote to avoid spending campaign energy on stay-at-home voters who may be persuaded to believe in the candidate, but who will not turn out regardless of political belief (P(vote) > .5).

As such, the underlying source credibility factors determine whether the voter is "open" to the candidate’s message (i.e. will update in the desired manner). Of the sub-group of (desirable) voters who fit this criterion, a random selection (the amount based on "reach") are selected for contacting. Both campaigns may contact the same voter multiple times during the simulation, but not more than once on a single "day".

In sum, voters entertain prior beliefs about each candidate, rate each candidate for trustworthiness and expertise, and have a signalling factor.

**Fig 2:**
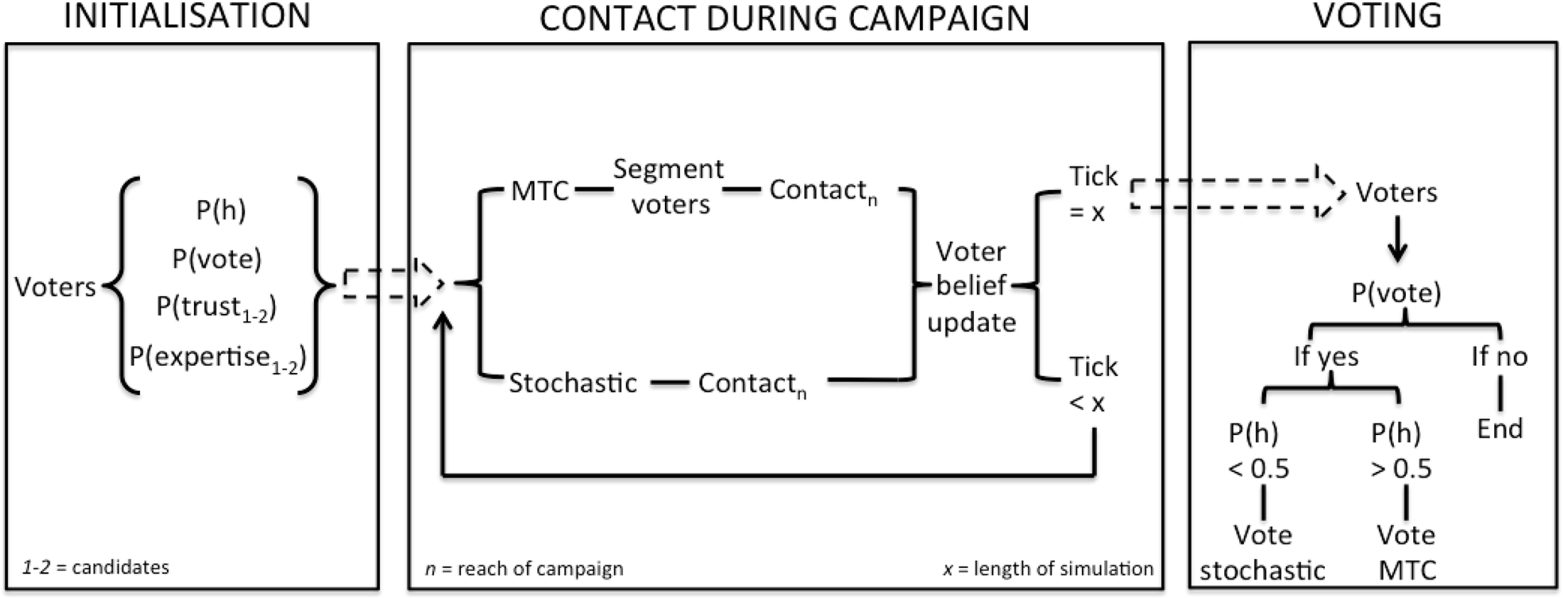
Model flow (spin-up phase, contact phase, and voting phase).

When a candidate contacts the voter, the voter updates the belief in p(candidate) in accordance with the Bayesian source credibility model. Candidates are either stochastic (non-MTC) or use the signalling factor to identify favourably disposed voters. Each candidate can reach a fixed number of voters each click. There are 10.000 voters and 2 candidates, and the campaign lasts for 50 days (ticks). Votes are cast at the end of each simulation (see Fig 2 for a depiction of the model flow).

## Simulation results and discussion

**Fig 3:**
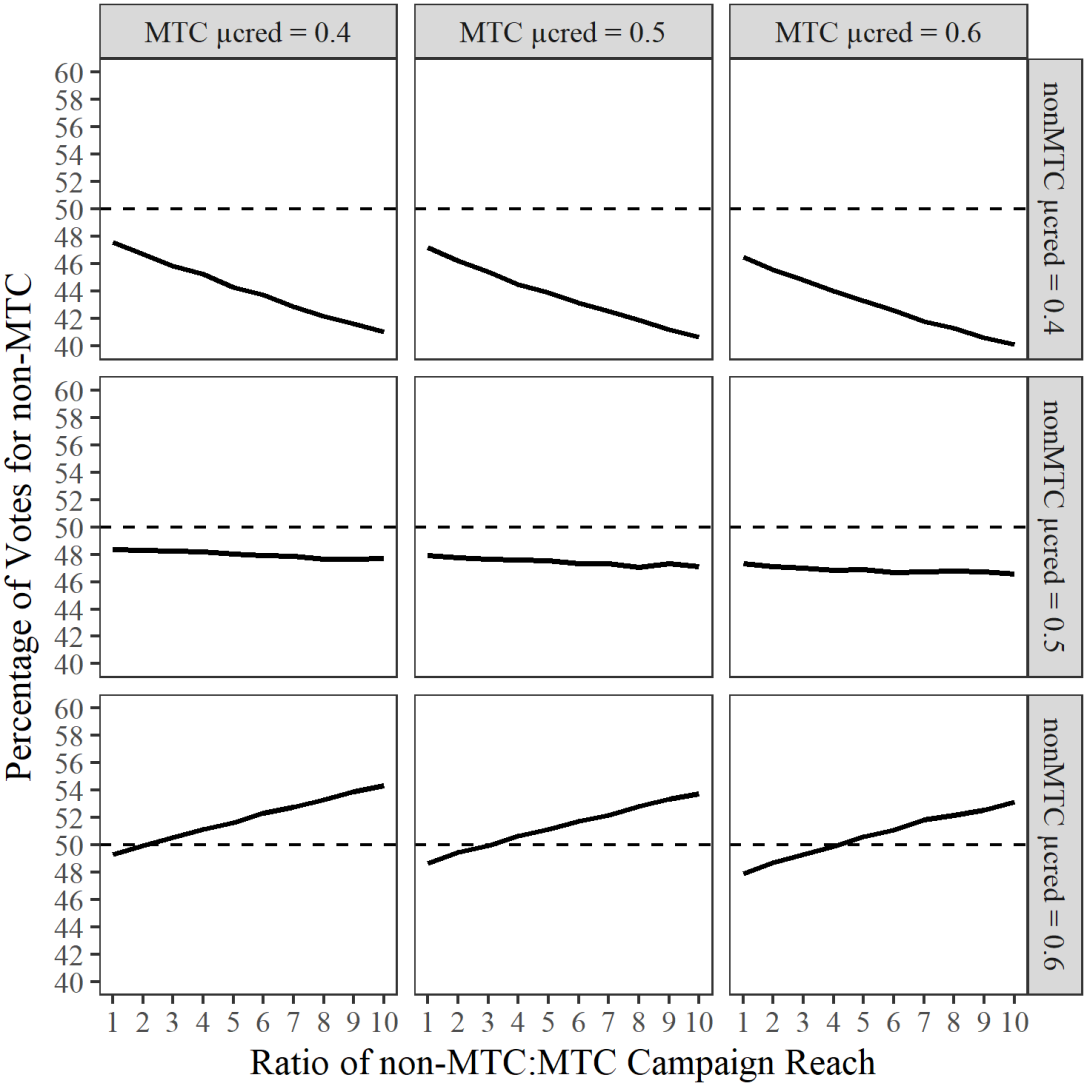
Voting outcome of simulated election campaigns. ^a^Dashed line represents break-even point between candidate vote shares.

The simulations demonstrate that MTCs are powerful strategic tools that give the candidate a substantial advantage compared with stochastic campaign strategies (S2 File). Given equal reach, the MTC candidate ‘won’ every election in all combinations of credibility assignments (see Fig 3). That is, even stochastic candidates that were significantly more credible required a more extensive reach to counter the MTC strategy.

We observe that MTCs are very effective in situations where both candidates are liked (μ_cred_ = 0.6). Here, the stochastic, 0.6, requires roughly 4.7 times larger reach to counteract the MTC effect (i.e. in the model the stochastic reaches 94 voters every “day” whilst the MTC reaches 20). Further, when the stochastic candidate has a credibility advantage (μ_cred_ of 0.6 to an MTC candidate μ_cred_ of 0.5) in the broad population, the stochastic candidate still requires roughly 2.2 times larger reach to counteract the MTC effect (bottom-centre facet, Fig 3). More interestingly, a disliked MTC candidate (μ_cred_ = 0.4) can always beat a moderately liked stochastic candidate (μ_cred_ = 0.5) by avoiding voters prone to backfire effects for the MTC candidate (left-hand facet of middle row, Fig 3).

Second, we observe an important deficiency in stochastic campaign strategies. If the candidate is disliked (e.g. μ_cred_ = 0.4), broader reach becomes a liability. This suggests that candidates with credibility deficiencies may hinder their chances by increasing visibility unless they can repair the credibility deficiency. This finding is important in times where some elections have featured candidates have been perceived negatively due to attack ads and negative campaigning. It suggests that campaigns can be self-destructive if they fail to perceive the population’s perception of the candidates’ credibility. If desperate about turning around a failing campaign, a candidate may increase visibility (e.g. by taking out adverts with broad appeal) with detrimental consequences.

Finally, the campaign suggests that in situations where *both* candidates are unpopular, MTC strategies are increasingly efficient, as the candidate can avoid targeting voters who are negatively disposed towards the candidate (and thereby avoid the above-mentioned self-destruction). In other words, the MTC does not waste time or make backfire mistakes. In situations with equally unpopular candidates (e.g. μ_cred_ = 0.4), we should expect MTC candidates to galvanise their base rather than reaching across the middle to engage with voters from the other political spectrum. MTC strategies would allow maximisation of efficiency in building the electoral base of the candidate, as the MTC candidate would be able to identify and engage with voters who happen to be favourably disposed. Further, in situations with disliked candidates, we believe MTC candidates can better gain swing voters (identifying swing who have a favourable view of the candidate in question). Indeed, if political apathy is high, low credibility of both candidates may depress voter turnout [30], making it increasingly efficient to galvanise one’s own base. This is partly due to the fact that depressed voter turnout yields scarcity of available voters, meaning that each vote becomes proportionally more valuable. Given the above, we predict MTCs to be highly useful in antagonistic campaign scenarios where both candidates are disliked (such as was reported in the 2016 election in the USA).

While the voter model relies solely on the voters’ perception of the credibility of the candidate, the method provides a tool for exploring more complex voter models. We consider some of these extensions below.

## Future directions

The present paper uses Agent-Based Modelling as a novel method to test the expected efficiency of specific campaign strategies by imbuing the voter in the model with cognitive architecture. To implement this, we focused on source credibility, as studies show perception of credibility influences reasoning and voting intention (see [10] for reasoning; [30] for voting). The paper provides a methodological proof of principle, from which more complex models of campaign strategies, as well as simulations of actual campaigns, can be explored (e.g. the 2016 presidential election in the US). In the following, we consider a few extensions we believe will provide fruitful avenues of research.

### Voter complexity

While the current model is computationally ‘clean’ and Bayesian, the real world will be messier and less organised. Given that MTC candidates gain a strategic advantage from data about the electorate, it is plausible that increasingly complex voters (e.g. with policy-based political preferences, different personalities, social connectivity, etc.) would gain even more from targeted strategies. If our predictions are correct, MTCs will be increasingly effective given a messier world with higher voter complexity if they are backed by big data and machine learning to effectively sift through the complexity for the variables of interest. This also suggests that an MTC with a poor or wrong model of voters are more liable to commit strategic mistakes. That is, the success of MTC strategies depend on the goodness of the model implemented in the MTC. Given a good model of the electorate (as is the case in the current model), the current paper presents the minimal efficiency of MTCs.

### System complexity

The present paper explores the relative efficiency of MTCs in political campaigns. However, the methodology used may be broadened to explore the efficiency of MTC strategies in other types of campaigns, such as public health initiatives and recycle campaigns, both of which are continuous rather than limited in time. Further, while voters in the model consider one hypothesis, representing binary elections (e.g. US presidential elections, the Brexit referendum), many elections feature multiple candidates and parties (e.g. party primaries in the US, elections in most democratic systems).

## Concluding remarks

We show that MTC candidates regularly beat stochastic candidates, even in situations where the MTC candidate is moderately disliked (μ_cred_ = 0.4) and the stochastic candidate is moderately liked (μ_cred_ = 0.6). In all simulations, the stochastic candidate required a reach that was, minimally, 2.2 times more extensive than the MTC in order to counteract the strategic gain of voter targeting. In races where both candidates are disliked, MTC strategies are highly effective and, in the current model set-up, even unbeatable. In these instances, the MTC persuader will predominantly galvanise and build a political base, as they can more efficiently target persuadable voters from the centre (e.g. identifying swing-voters who have a favourable view of the candidate in question), whereas a stochastic campaign would appeal more broadly and indiscriminately to swing voters.

The effectiveness of an MTC depends on a few key parameters. First, the quality of voter data that is available to the MTC candidate; if the data is noisy, the MTC candidate might wrongly classify voters. Second, the reach of the campaign; campaigns become more effective if they can reach more voters per day. We further believe the efficiency of MTCs will increase when voter complexity increases while effects may vary given greater system complexity. In elections with relative differences in electoral voting power (e.g. swing states, gerrymandering, first-past-the-post, etc.), we predict that micro-targeted strategies will be increasingly efficient. For example, a voter may be entirely persuadable (and thus susceptible to a targeted engagement). However, if the voter, living in a first-past-the-post system, inhabits a ‘safe seat’ (i.e. a seat that always votes Republican), the persuasive effort, though successful, would be an inefficient allocation of campaign resources.

While MTCs do not guarantee victory, as there are always variables outside of a campaign’s control (e.g., economic shocks, uncovered scandals, etc.), they appear to lend a critical advantage to the candidate in question. If the MTC has access to big data, computing power to detect and process relevant signals within the data, and if the campaign works from models that describe and predict factors relevant to voting intention and behaviour, increased voter complexity should yield serious strategic advantages. This points to a potential democratic problem. MTCs invariably cost a significant amount of money to run, as the campaign requires data, analysts, and dynamic campaign management. If one campaign has a financial advantage, this may lead to an unassailable advantage. This suggests a critical need in deliberative democracies to consider campaign funding, data accessibility, and openness of campaign management. If deliberate and targeted misinformation can be interjected into the population, this requirement only increases.

In sum, the model shows that MTCs are a highly efficient way of managing campaigns in dynamic environments, as they allow for adaptive strategies relative to broad campaigning. As MTCs become increasingly prevalent in politics and other campaigns, it is crucial to understand the mechanism and efficiency of these models. Imbuing the agents with cognitive principles provide an initial proof of methodological concept that MTCs can be modelled and understood using agent-based models. We believe this will be an important area of research in cognitive, social, and political sciences for years to come.

## Supporting information

S1 File ODD+D protocol. (DOCX)

S2 File Simulation data. (CSV)
