# Supplementary material for: A method for evaluating cognitively informed micro-targeted campaign strategies: An agent-based model proof of principle
Source: PLoS One. 2018 Apr 10;13(4):e0193909. doi: 10.1371/journal.pone.0193909 (PMC5892896; doi:10.1371/journal.pone.0193909)
Supplement: S1 File — (DOCX) [file pone.0193909.s001.docx]

**ODD+D Protocol**

**Title: A method for evaluating cognitively informed micro-targeted campaign strategies: An agent-based model proof of principle**

**I) Overview**

*Purpose*

Recent years have seen an increase in micro-targeted campaigns (MTCs) in political campaigns. These involve data-rich models of individual voters, which allows for optimisation of persuasion strategies. While MTCs have been roundly identified qualitatively and anecdotally, political psychology lacks a formal method for describing and testing various MTC strategies.

By developing a formal method for describing and evaluating effectiveness of MTCs, we provide a foundational tool for researchers who want to explore specific elections (which involves empirical grounding and parameter fitting), explore the impact of specific cognitive models (comparison of outcomes), explore the efficiency of different types of MTC strategies (hypotheticals), lessen the influence of wilful misinformation, among many others. The model provides a method for exploring these complex systems using cognitively informed voters and politicians.

Additionally, elections are innately complex phenomena, as politicians may engage with voters, voters may engage with other voters, and voters may engage with news outlets, and so forth. The current framework provides a method that is easily scalable to encompass this increased interactivity (e.g., the use of social networks).

In sum, the purpose of the paper is twofold. One, to provide a formal method for exploring and evaluating the impact of cognitively informed MTCs. Two, to illustrate a naturally scalable framework that is capable of handling the complex questions posed by modern society. The model is predominantly designed for political, cognitive, and social psychologists, political scientists, and philosophers, but can be used by anyone interested in cognition, information flow, and campaign governance and management (political or otherwise).

*Entities, state variables, and states*

The basic model has three entities: stochastic and micro-targeting persuaders (two types of political campaign for a candidate), and voters. Voters have stochastically assigned credibility estimates of each political candidate (perceived trustworthiness and expertise between 0 and 1, see ‘initialisation’). These estimates determine their reaction to persuasive attempts (see ‘learning’).

Stochastic persuaders have no explicitly held belief states, but may engage persuasively with voters (see ‘individual sensing’) – that is, they do not hold beliefs personally, but transmits reports about the belief that voters entertain. In doing this, they convey their message (see ‘interaction’) and the voter updates its beliefs (see ‘learning’). MTC persuaders are identical to stochastic persuaders with one exception: they filter out voters who are negatively disposed towards their candidate (see ‘interaction’). As such, they also have no explicitly held belief states.

Voters have three classes of belief states: belief in the hypothesis (“Which candidate am I voting for?”), belief in the trustworthiness of each candidate (the model requires a minimum of 2 persuaders), and belief in the expertise of each candidate.

There are no geographical features in the current model. Voters are distributed stochastically and reach does not depend on candidate proximity. While the geography might be a relevant factor in actual elections (e.g. the capacity to reach a small village), the current design mirrors an online network where people can contact and be contacted regardless of geographical distance. Introducing geographical limitations, however, is easily scalable within the current framework. Likewise, the basic model (in the current paper) does not have social network structures between voters, but only has links between persuaders and persuadees. The lack of social structure between voters was chosen to explore a minimal model. Like geography, the social networks and interactions between voters is a scalable feature that can easily be integrated within the current framework. As such, the current framework provides the foundational model for future explorations of more specific issues.

**II) Design concepts**

*Theoretical and empirical background*

The cognitive foundation rests on Bayesian belief revision (see e.g., Chater & Oaksford, 2007; Hahn et al., 2009; Harris et al., 2015), providing a normative foundation for reasoning. Further, the Bayesian approach to argumentation has captured fallacies (Corner et al., 2011; Harris et al., 2012) and argumentation more generally (Hahn & Oaksford, 2006; 2007). We chose this cognitive apparatus, as it is empirically supported and normative in nature. Crucially, though, voters’ cognitive components are modular such that competing revision mechanisms (normative or otherwise) can be implemented and tested. As with the geographical and network features, cognitive features are also scalable and replaceable.

Given that the current model is a proof of principle for a formal method for evaluating cognitively informed MTC strategies, there is little empirical grounding. However, the conditional probabilities that underpin the cognitive aspect of the model are empirically grounded, elicited by Madsen (2016), see ‘learning’. Also, on a broader level, the framework allows for model comparison against real data given implementation for actual elections. This, though, is an empirical challenge of specific models while the present model represents a framework from which these models can be developed and tested.

The background for the model rests on the identification of MTCs as a successful and efficient method of political campaigning (as well as in marketing more generally). While this has been identified qualitatively (e.g., Issenberg, 2012; Bimber, 2014), no formal model has yet been offered to test the relative efficiency of such campaign types. It is worth noting the proposed framework allows for quantitative comparisons between micro-targeted campaigns and more traditional campaigns that rely on broader (or no) segmentation.

*Individual decision-making*

Only voters make active decisions in the model (namely, at the end of the simulation, they choose to cast their vote for candidate A or B). The persuaders decide which voters with whom they wish to engage with persuasively, but this is classified as interaction, rather than decision-making (see ‘interaction’).

Each voter is outfitted with a given likelihood of voting (P(Vote); µ= 0.5, * *= 0.25, bounded between 0.01 and 1), drawn from a normal distribution. At the end of the simulation (after 50 ticks – ticks being arbitrary time point, e.g. 1 day), voters vote with that probability. If voters do decide to cast their vote, they do so on the basis of the final belief state of their hypothesis, P(H), regarding for which candidate to vote (i.e. the goodness of the candidates). If P(H) < 0.5, they vote for candidate A, if P(H) > 0.5, they vote for candidate B.

*Learning*

Voters update their beliefs in the goodness of each candidate through a Bayesian source credibility model (Harris et al., 2015). This amalgamates prior belief in the hypothesis, P(H), with perceived credibility (trustworthiness and expertise) as follows:

$$P(H|Rep)= \frac{P\left( H \right)P(Rep|H)}{P\left( H \right)P(Rep|H)+P\left( \neg H \right)P(Rep|\neg H)}$$

P(H|Rep) represents the probability that the hypothesis is true (H) given a confirming statement (Rep). P(H) represents the prior belief in the hypothesis, and P(Rep|H) and P(Rep|¬H) represent the conditional probability that the source would provide a positive statement if the hypothesis was indeed true/false. Trustworthiness and expertise are integrated within P(Rep|H) and P(Rep|¬H) through the combination of conditional probabilities. To calculate P(Rep|H) and P(Rep|¬H), we use:

P(Rep|H) = P(Rep|H, E, T) * P(E) * P(T) + P(Rep|H, ¬E, T) * P(¬E) * P(T) + P(Rep|H, ¬E, ¬T) * P(¬E) * P(¬T) + P(Rep|H, E, ¬T) * P(E) * P(¬T), *mutatis mutandis* for P(Rep|¬H)

This integrates credibility estimates such that highly credible sources yield greater adherence with the proposed hypothesis. Conditional probability estimates are taken empirically from Madsen (2016).

|  | T, E | T, ¬E | ¬T, E | ¬T, ¬E |
| --- | --- | --- | --- | --- |
| H | 0.80 | 0.58 | 0.34 | 0.18 |
| ¬H | 0.22 | 0.42 | 0.59 | 0.71 |

Table 1: conditional probabilities

It should be noted that as P(H) reflects the singular dimension across two candidates (i.e. when P(H) < 0.5, voter favours candidate A, whilst when P(H) > 0.5, voter favours candidate B), to handle persuasive attempts at decreasing P(H) (i.e. to persuade a voter of candidate A), P(H) is inverted to run through the model, and reverted after. In this way, the model handles positive persuasion in either direction.

Lastly, persuaders have no learning, as they merely attempt to convince the electorate to vote for them at the end of the simulation.

*Individual sensing*

The voters are receptive agents who do not actively seek out anything – thus, they do not sense per se. The candidates can locate voters with certainty and connect with individual voters. They can do so over the entire physical space of the simulation (that is, Euclidean distance from candidate to voter does not matter). The MTC candidate senses the belief states of each voter and disregards undesirable connections (see ‘interaction’). There are no errors in candidate sensing.

*Individual prediction*

Individual voters use their beliefs to guide their eventual voting (see ‘individual decision-making’) and the MTC candidate uses these beliefs to select for most eligible voters (see ’interaction’). Other than this, agents do not predict the future.

*Interaction*

The interaction in the current model is between active persuaders (stochastic and MTC politicians) and passive persuadees (voters). For each tick, the persuaders establish contact with *n* voters, defined as ‘candidate reach’. In the simulations, the MTC candidate has a fixed reach of 20, whilst the reach of the non-MTC candidate is manipulated to test the efficiency of the MTC strategy. This contact generates links between the persuader and *n* persuadees.

Each voter has a perceived credibility score for each candidate, P(Cred_A_) and P(Cred_B_), which is the average of the voters perceived trust (P(T_A_) and P(T_B_)) and perceived expertise (P(E_A_) and P(E_B_)) in each candidate. The MTC segments voters and only contacts those who are not already strongly for or against the candidate, and perceive the candidate as credible, and likely to vote. That is, P(H) > .25 & < .75, P(Cred_Candidate_) >= .5 and P(Vote) > .5. The stochastic candidate does not segment voters, but contacts *n* voters each tick entirely at random. As candidates contact voters, candidates transmits reports pertaining to the hypothesis (see ‘learning’).

*Collectives*

No collectives are formed in the model.

*Heterogeneity*

Agent heterogeneity arises not only from the three different classes of agents that exist within the simulation (voters, the stochastic candidate, and the MTC candidate), but is also generated within the voter class. As described in ‘initialisation’, voters are spawned with heterogeneous beliefs about the candidates, their credibility, and the likelihood of voting.

*Stochasticity*

Agent beliefs and voting likelihood are all stochastically assigned at the point of initialisation (see ‘initialisation’).

*Observation*

The key output of the model is the eventual result of the election. At the end of the simulation, voters cast a vote with their respective probability of voting, P(Vote). If they do vote, they use their belief state concerning the hypothesis of candidate preference. If their subjective belief in the hypothesis, P(H), < 0.5, they vote for candidate A; if P(H) > 0.5, they vote for candidate B. This output can be used to calculate the relative efficiency (i.e. strategic advantage) of implementing a particular micro-targeted campaign.

**III) Implementation details**

*Initialisation*

The model spins up by generating 10.000 voters, each of which has three classes of beliefs. Each voter is outfitted with a voting likelihood, P(Vote); µ= 0.5, * *= 0.25, bounded between 0.01 and 1), drawn from a normal distribution. Further, each voter is outfitted with a prior belief in the hypothesis, P(H); µ= 0.5, * *= 0.25, bounded between 0.01 and 1), drawn from a normal distribution. Finally, each voter assigns perceived trust and expertise of each candidate, P(T_A_), P(T_B_), P(E_A_) and P(E_B_); (all µ= 0.5, * *= 0.25, bounded between 0.01 and 1), drawn from normal distributions.

*Input data*

The model does not use any external data, as it is a proof of methodological principle.

*Submodels*

There are no submodels in the current version of the MTC ABM.
